# Ebola Virus Genome Plasticity as a Marker of Its Passaging History: A Comparison of *In Vitro* Passaging to Non-Human Primate Infection

**DOI:** 10.1371/journal.pone.0050316

**Published:** 2012-11-28

**Authors:** Jeffrey R. Kugelman, Michael S. Lee, Cynthia A. Rossi, Sarah E. McCarthy, Sheli R. Radoshitzky, John M. Dye, Lisa E. Hensley, Anna Honko, Jens H. Kuhn, Peter B. Jahrling, Travis K. Warren, Chris A. Whitehouse, Sina Bavari, Gustavo Palacios

**Affiliations:** 1 Genomics Division, United States Army Medical Research Institute of Infectious Diseases (USAMRIID), Fort Detrick, Maryland, United States of America; 2 Virology Division, United States Army Medical Research Institute of Infectious Diseases (USAMRIID), Fort Detrick, Maryland, United States of America; 3 Toxicology Division, United States Army Medical Research Institute of Infectious Diseases (USAMRIID), Fort Detrick, Maryland, United States of America; 4 Diagnostic Systems Division, United States Army Medical Research Institute of Infectious Diseases (USAMRIID), Fort Detrick, Maryland, United States of America; 5 Integrated Research Facility at Fort Detrick, National Institute of Allergy and Infectious Diseases, National Institutes of Health, Fort Detrick, Maryland, United States of America; 6 Simulation Sciences Branch, United States Army Research Laboratory, Aberdeen Proving Ground, Maryland, United States of America; Metabiota, United States of America

## Abstract

To identify polymorphic sites that could be used as biomarkers of Ebola virus passage history, we repeatedly amplified Ebola virus (Kikwit variant) *in vitro* and *in vivo* and performed deep sequencing analysis of the complete genomes of the viral subpopulations. We then determined the sites undergoing selection during passage in Vero E6 cells. Four locations within the Ebola virus Kikwit genome were identified that together segregate cell culture-passaged virus and virus obtained from infected non-human primates. Three of the identified sites are located within the glycoprotein gene (*GP*) sequence: the poly-U (RNA editing) site at position 6925, as well as positions 6677, and 6179. One site was found in the VP24 gene at position 10833. In all cases, in vitro and in vivo, both populations (majority and minority variants) were maintained in the viral swarm, with rapid selections occurring after a few passages or infections. This analysis approach will be useful to differentiate whether filovirus stocks with unknown history have been passaged in cell culture and may support filovirus stock standardization for medical countermeasure development.

## Introduction

Filoviruses (family *Filoviridae*) are etiological agents of severe viral hemorrhagic fever in humans and non-human primates with case-fatality rates ≈25–90%. The family *Filoviridae* includes two genera, *Ebolavirus* and *Marburgvirus*, to which five ebolaviruses (Bundibugyo, Ebola, Reston, Sudan, and Taï Forest) and two marburgviruses (Marburg and Ravn) are assigned, respectively [1], [2]. Importantly, while there are five different ebolaviruses assigned to five separate species, there are only two marburgviruses, and both are assigned to a single species. A third tentative genus “Cuevavirus” has been suggested for the newest member of the family, Lloviu virus [3].

In the United States, filoviruses are classified as Select Agents [4], NIH/NIAID Category A Priority Pathogens [5], and CDC Category A Bioterrorism Agents [6] due to the absence of FDA-approved prophylaxis or treatment regimens, their high infectivity, and their stability in aerosols [7]. Consequently, the development of medical countermeasures (MCM), such as antivirals or vaccines, is a high priority for biodefense. In this context, the majority of ebolavirus research was performed using Ebola virus (EBOV). More importantly, the majority of modern MCM evaluations and the characterization of pathogenesis in non-human primates (NHP) was performed with one particular variant of EBOV [8], Kikwit (EBOV-Kik), isolates of which were obtained during a large outbreak of Ebola virus disease in Kikwit, Zaire (now Democratic Republic of the Congo) [9].

Recently, studies from Volchkova *et al.* demonstrated the existence of genomically stable mutations in the EBOV genome that appear to be related with its passaging history. The identified changes were predominantly found in the glycoprotein (*GP*) gene [10]. In contrast to the marburgvirus *GP* gene, which consists of a single open reading frame (ORF) and expresses a single glycoprotein (GP_1,2_), the ebolavirus *GP* gene expresses three products (GP_1,2_, sGP, and ssGP). This is achieved by the alternative use of three overlapping ORFs. In particular, the expression ratio of the three proteins is regulated via a stretch of seven uridylyls (7U) commonly referred to as the mRNA editing site. During replication in the host cell, a soluble glycoprotein of unknown function (sGP) is the primary expression product of the *GP* gene. GP_1,2_ is expressed only when an extra (eighth) adenylyl is inserted into the nascent mRNA via stuttering of the EBOV RNA-dependent RNA polymerase over the editing site. Likewise, ssGP, another soluble glycoprotein of unknown function, is produced when the polymerase adds two nontemplate adenylyls (or ignores a template U) [11], [12]. It has been reported that sGP constitutes ∼75% of the glycoprotein expressed during infection [13], [14]. GP_1,2_ is a structural protein that forms trimers and localizes to the Ebola virion membrane. It is primarily responsible for attachment of the virion to its host cell-surface receptor and subsequent fusion [15]. The glycoprotein sGP is a nonstructural secreted protein. Volchkova *et al.* demonstrated that serial passage of a recombinant EBOV (variant Mayinga; EBOV-May) containing a wild-type (7U) editing site in Vero E6 cells resulted in a viral population that predominantly contained an 8U editing site. However, when 8U virus was injected into guinea pigs, the population became predominantly 7U [10]. The authors suggested that these changes were related to selective advantages linked to the controlled expression of GP_1,2_ and/or sGP. This study was targeted exclusively to the *GP* gene mRNA editing site and was performed using traditional clonal analysis.

For our study, we used deep sequencing to characterize the entire EBOV-Kik genome during viral passage in cell culture and after infection of NHP, one of the standard animal models for filovirus infection and currently the most highly regarded model for MCM research and development. The aim of the study was to determine whether the results of Volchkova *et al.* are variant- and/or animal-specific and whether hotspots other than the mRNA editing site exist in EBOV subpopulations. This is an area of particular interest with the increasing use of animal data for the approval of therapeutics for human use when human trials are not possible. We focused the study on the interface between in vivo infection and in vitro propagation of viral stocks.

## Materials and Methods

### Viruses

**Figure 1:**
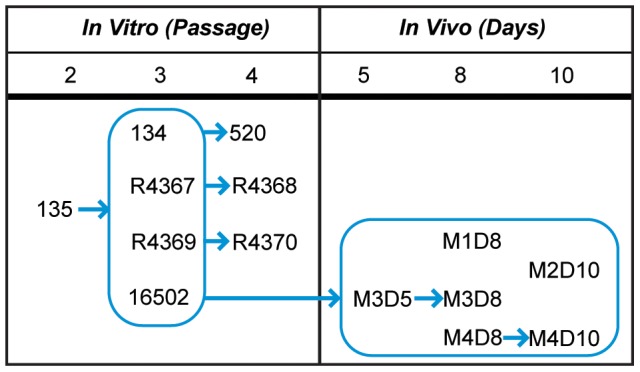
EBOV-Kik sample history is modeled here over passaging and infection time. Arrows indicate parent child relationship of the isolates and infections (e.g., M1D8 was derived by infecting an NHP with passage 3 stock 16502 which was derived from passage 2 stock 135). All passage 3 virus were obtained from 135. Passage 4 virus were derived from the preceding stocks (indicated by an arrow). All “in vivo” samples were derived after infection with viral stock 16502. Where multiple days were available from the same animal an arrow describes the progression of samples.

All work with infectious Ebola virus (species *Zaire ebolavirus*, genus *Ebolavirus*, family *Filoviridae*, order *Mononegavirales*) was performed at the United States Army Medical Research Institute of Infectious Diseases (USAMRIID) at Fort Detrick, Frederick, MD, USA within maximum (biosafety level 4) containment. We used the only Kikwit isolate of Ebola virus (EBOV-Kik) available at USAMRIID, isolate 9510621 – this isolate had been used for all major USAMRIID NHP studies. EBOV-Kik 9510621 was originally isolated at the CDC Special Pathogens Unit from a sample taken from a female patient who died during the 1995 Ebola virus disease outbreak in Kikwit, Zaire (now Democratic Republic of the Congo). According to available records, the first and second passages of the virus were performed in grivet (*Chlorocebus aethiops*) kidney epithelial Vero E6 cells (ATCC #CRL-1586) to produce an initial virus stock, designated here as #135 (passage 2). After an additional passage in Vero E6 cells, the virus, designated #134 (passage 3), was sequenced at USAMRIID using classical methods by Chain *et al.* and Ichou *et al.* Near-complete genomic sequence (lacking 3′ and 5′ UTRs) was deposited in GenBank (accession # AY354458). Importantly, classical sequencing revealed an 8U mRNA editing site. We used three independent lineages of virus adaptation after passaging in Vero E6 cells from the original passage 2 stock, 135. Figure 1 contains the passage histories for these viruses and Table 1 contains a list of alternate identifiers used for the different stocks. Two separate animal studies conducted at USAMRIID (AP-09-033 and AP-10-014) provided six viral samples obtained from the blood of four crab-eating macaques (*Macaca fascicularis*). Samples were harvested on days 5, 8, or 10 from animals challenged with viral stock 16502 (passage 3). These samples are referred to as M1D8, M2D10, M3D5, M3D8, M4D8, and M4D10. All viral samples were obtained from mock-treated animals; only samples with viral burden above ∼100,000 gc/ml were included.

**Table 1:** Stock identifier alias table.

Stock ID*	CDC#	AIMS#	RIID#	Passage#
135	807224	135 and 10120		2
134		134		3
R4367		22327	R4367	3
16502		16502		3
R4369		22882	R4369	3
520		520		4
R4368		22433	R4368	4
R4370		22955	R4370	4

* Stock ID is used to identify the stock in the text.

### Animal Use Statement

Research was conducted under an IACUC approved protocol in compliance with the Animal Welfare Act, PHS Policy, and other Federal statutes and regulations relating to animals and experiments involving animals. The facility where this research was conducted is accredited by the Association for Assessment and Accreditation of Laboratory Animal Care, International and adheres to principles stated in the Guide for the Care and Use of Laboratory Animals, National Research Council, 2011. The Institutional Animal Care and Use Committee of the United States Army Medical Research Institute of Infectious Diseases approved these studies. Animals were individually housed in stainless steel cages and were provided food and water ad libitum. Animal rooms were maintained on a 12-h light/dark cycle and the animals were provided toy and fruit environmental enrichments. Animals were monitored at least twice daily for signs of distress. Buprenorphine was administered to animals displaying clinical signs of discomfort and meloxicam was administered to animals exhibiting elevated body temperature. Euthanasia was performed to minimize pain and distress by intravenous administration of sodium pentobarbital.

### Sequencing

RNA was extracted using Trizol LS (Invitrogen, Carlsbad, CA) and used for cDNA synthesis by sequence-independent single primer amplification (SISPA) [16]. First-strand synthesis was performed with the Superscript III first-strand synthesis system (Life Technologies/Invitrogen, Carlsbad, CA) and a primer containing random hexamers at the 3′ end. The second strand was synthesized by adding Klenow (3′→5′ exo-) DNA polymerase (New England Biolabs, Ipswitch, MA). cDNA was purified, and then amplified with MyTaq DNA polymerase (Bioline, Tauton, MA). After purification with the MinElute PCR purification kit (QIAgen, Valencia, CA), PCR products were fragmented using the Covaris S2 instrument (Covaris, Woburn, MA). Libraries were prepared with the Illumina TruSeq DNA sample Preparation kit (Illumina, San Diego, CA), according to the manufacturer's protocol. The libraries were evaluated for quality using the Agilent 2100 Bioanalyzer (Agilent, Santa Clara, CA). After measurement by real-time PCR with the KAPA qPCR Kit (Kapa Biosystems, Woburn, MA), libraries were diluted to 10 nM. Cluster amplification was performed on the Illumina cBot and libraries were sequenced on the Illumina GAIIx using the 76 bp paired-end format.

### Analysis

Viral assemblies were completed in DNAStar Lasergene nGen (Madison, WI) with ≈4×10^5^ reads. Amplification (SISPA) primer removal, quality trimming, and trim-to-mer were performed on reads with a minimum similarity of 93% (four base mismatch). Single-nucleotide polymorphisms (SNPs) with fewer than 200 read depth were removed from the analysis. Only SNPs present in the population above the 2% threshold are presented in this report. A consensus change is defined here as a change relative to the published sequence for EBOV-Kik (GenBank accession # AY354458) present in ≥50% of the population. Below that threshold, SNPs are considered subclonal substitutions and part of a minority subpopulation of the virus. Because the sequence is derived by random hexamer amplification using the SISPA protocol, these results do not distinguish between genomic and messenger RNA.

## Results and Discussion

Near-complete genome reconstruction was achieved for all EBOV-Kik passages and for viruses in NHP serum samples. The assemblies generated were at a depth of ≈1,000 and coverage of >99% for all specimens (Table 2). For purposes of the analysis, GenBank sequence AY354458 (EBOV-Kik), derived from Sanger sequencing of #134, was used as the reference sequence for stocks 134, 135, 520, 16502, R4367, R4368, R4369, R4370, and the serum samples (M1D8, M2D10, M3D5, M3D8, M4D8, and M4D10). There was only one mutation detected when comparing all passages with the GenBank reference strain # AY354458; a mutation observed at non-coding position 7327 (C to T).

**Table 2:** Mutation and SNPs analysis of EBOV passage variants.

					Pass 2	Pass 3				Pass 4			Day 5	Day 8			Day 10	
Ref Pos	Codon	AA	NT	Gene	135	R4369	134	16502	R4367	520	R4370	R4368	M3D5	M1D8	M3D8	M4D8	M4D10	M2D10
3412	T:ACT @ 95−>N:AaT	95	284	VP35	7.4%	6.4%	1.4%	<1.0%	5.8%	2.2%	4.0%	1.8%	<1.0%	4.0%	<1.0%	<1.0%	<1.0%	3.9%
6179	E:GAG @ 47−>D:GAt	47	141	GP	37.2%	11.7%	20.8%	7.7%	8.7%	9.9%	7.0%	9.9%	29.7%	47.5%	67.2%	65.0%	91.2%	35.7%
6677	Y:TAC @ 213−>.:TAa	213	639	GP	2.0%	2.2%	4.7%	4.9%	5.0%	4.8%	2.6%	4.3%	5.5%	1.6%	3.0%	4.8%	3.7%	1.0%
6925	K:AAA @ 295−>X:AAx	295	885	GP	33.7%	16.5%	17.9%	2.1%	1.6%	8.1%	7.6%	7.4%	72.2%	96.8%	62.6%	56.9%	73.4%	99.5%
7327					99.6%	99.2%	99.2%	99.0%	99.5%	98.6%	98.6%	99.8%	96.7%	98.4%	98.4%	96.4%	96.8%	99.6%
8003	G:GGT @ 655−>G:GGg	655	1965	GP	23.3%	27.3%	15.1%	11.1%	19.7%	13.2%	27.5%	16.6%	7.8%	18.8%	12.8%	14.8%	13.2%	15.8%
10833	R:AGA @ 163−>K:AaA	163	488	VP24	37.4%	10.4%	15.0%	4.5%	4.4%	8.6%	3.7%	3.2%	20.3%	45.8%	59.6%	62.0%	89.8%	25.5%
13987	G:GGA @ 802−>G:GGA	802	2406	L	<1.0%	4.3%	2.3%	2.7%	3.7%	2.7%	3.8%	2.1%	9.1%	2.4%	4.8%	4.6%	4.5%	1.1%

**Figure 2:**
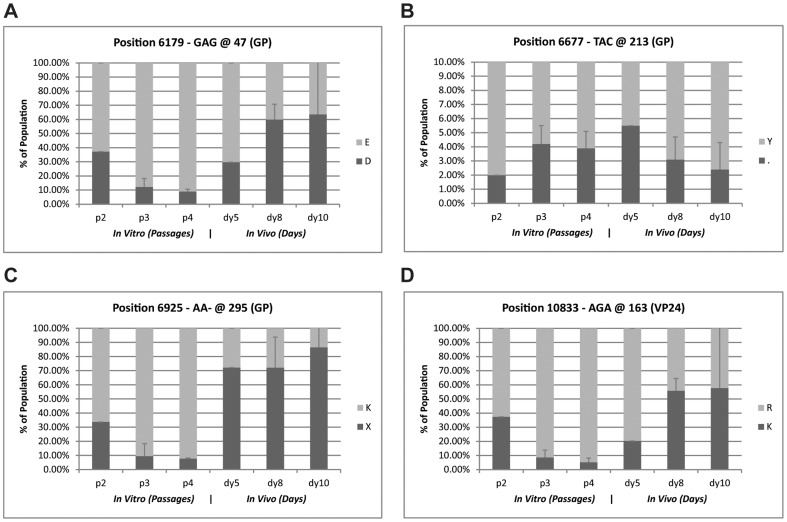
Comparison of EBOV-Kik *in vitro* passage with *in vivo* infection. EBOV-Kik samples from passage 2, 3, and 4 in Vero E6 cells (represent 1, 4 and 3 isolates respectively) were compared to six in vivo samples from lethally challenged crab-eating macaques collected after day 4. A passage 3 viral stock from this study (16502) was used as the challenge material for the *in vivo* samples. See Figure 1 for passage and seed stock information. Numbers are reported here as percent of population for sub-clonal variants. Error bars represent variance between multiple independently propagated lineages or infected animals (i.e. The data is summarized based on passage number or infection day rather than experimental or individual bounds). a) GP Poly-U transition. Here, we compare two variants of the 8U form that expressed predominantly the full length GP_1,_ and the 7U variant that predominantly expressed sGP. *In vitro*, we observed a dramatic and rapid shift between the 7U variant and the 8U variant at positions 6,925 and 6,926. By passage 3, there is an inversion in variant levels and there is an equally rapid reversion observed by day 8 *in vivo*. b) Stop Codon detection. There is a twofold increase in the amount of the sub-clonal variant encoding for a truncated form of GP_1,2_ at position 6,677.* Note: the scale is changed to 10% for better visualization. c) and d). Marker increase *in vivo*. We identify two changes 6,179, and 10,833, which result in amino acid changes _2_ protein in GP_1,2_ and VP24 respectively. As with the 7U variant, the subclonal variants at these positions decrease and revert rapidly when switching between cell culture passage and infection.

Our results confirm and expand the observations of Volchkova *et al.* We observed that, indeed, the EBOV-Kik 8U variant was the preferred state *in vitro* (passages from Vero E6 cells), whereas the 7U variant was the preferred state *in vivo* (virus obtained from infected macaques) (**Figure 2a**). Mutation of the mRNA editing site occurred rapidly, mostly in the first two passages in cell culture). We observed a dramatic and rapid shift at positions 6,925 and 6,926 *in vitro*; by passage 3 (stock 16502), there was an inversion of direction of change. An equally rapid reversion was observed when this passage 3 (16502) stock was utilized to infect macaques. At day 5, the earliest available time point we were able to sequence, the reversion to the 7U variant was nearly complete. These findings provide evidence that the 7U variant is not only selected for in guinea pigs, as shown by Volchkova *et al.*, but also in NHP, suggesting that a 7U mRNA editing site is a determinant for viral fitness in mammalian hosts. This hypothesis is further substantiated by the fact that the limited human-derived ebolavirus sequences deposited in GenBank to date, with the exception of EBOV-Kik, are 7U viruses. It remains unclear why the described selection takes place. A 7U to 8U switch likely reverses the expression ratio of sGP: GP_1,2_ from ≈80∶20 to ≈20∶80. An obvious conclusion is that during replication *in vivo* there is a strong selective pressure for the virus to avoid overexpression of GP_1,2_ and thereby the maintenance of a 7U mRNA editing site, although it cannot be ruled out that expression of sGP, whose function is still unknown, drives the selection. GP_1,2_ has been described by some groups to be cytotoxic, and indeed Volchkov *et al.* have suggested that mRNA editing is a mechanism to control the protein's cellular concentration [12], [17]. Conversely, sGP has been suggested to act as an anti-GP_1,2_ antibody decoy *in vivo* – low plasma sGP concentrations could therefore mean that more virions are neutralized, which of course would be detrimental to the virus [18].

**Figure 3:**
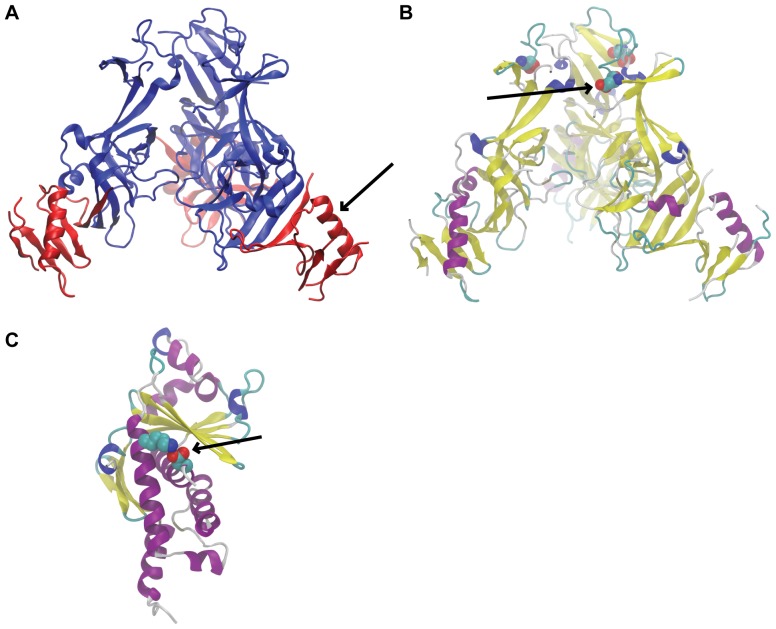
Predicted protein conformational changes. Locations of the observed mutations within the structures of EBOV proteins: a) GP truncations – occur right after a disordered region in the crystal structure. The truncation also removes the ends of the trimer (in red). b) GP_1,2_ – residue E47 (vdW spheres) is near the top of the trimer and, in the X-ray crystal structure (3CSY), makes no interactions with other side chains. c) VP24 – residue K163 forms a tight salt-bridge with D104 on another helix near an undetermined loop in the X-ray structure.

We have also identified during the course of this study a stop codon site within the *GP* gene at position 6,677 (**Figure 2b**). We observed a twofold increase in the amount of the sub-clonal variant encoding for a truncated form of GP_1,2_ during *in vitro* passaging and a modest reversion effect *in vivo*. It is plausible to envision this variant to function in a similar way to the 7U/8U variant, encoding for a non-functional version of GP_1,2_ as a laboratory passage adaptation to control full-length GP_1,2_ expression level. The truncation in the protein occurred right after a disordered region in the crystal structure of the GP_1,2_ and removed the end of the molecule (**Figure 3a**). To be coherent with the above GP/sGP equilibrium model, the truncated part of the molecule would need to carry the cytotoxic and/or decoy function. Nevertheless, it is noteworthy that two of the observed changes are mapped to the *GP* gene. Interestingly, this result, even when it is unexpected, it is not completely novel. A study of partial EBOV sequences recovered directly by PCR from wild apes demonstrated that the circulating consensus sequence of GP contained a stop codon at amino acid position 481 [19]. It seems clear that ebolaviruses evolution had resulted in a wide array of measures to control GP expression.

Complete EBOV-Kik genome analyses also revealed several other instances of changes that segregate between *in vitro* and *in vivo* virus populations. We found two additional sites with greater than twofold changes *in vitro*, which subsequently reverted to levels similar to early passage data when used in infection. None of these sites approached mutation levels (100% of population) yet interestingly, the direction of change was reverse in these cases (i.e., the diversity at these sites is higher in *in vivo* than *in vitro* passages: the population becomes more homogeneous during cell passaging). The first change was observed again in the *GP* gene (**Figure 2c**) and converts GP_1,2_ residues Glu47 to Asp. This residue does not make salt-bridge contacts in the wild-type crystal structure (PDB ID: 3CSY) [20], but there could be trimer bending motion in a biologically relevant environment which would bring this residue into contact with other trimer residues (**Figure 3b**). Thus, the E→D variant could have an effect on the range of this bending motion. This residue change is located in the area between the GP_1,2_ signal peptide (residues 1–32) and the receptor binding site of the *GP* gene (residues 54–201) [21]. No major functions have been mapped to this area, with the exception of a region that demonstrated a suppressive effect on T-cell proliferation *in vitro* using EBOV-May [22].

The second change was found in the *VP24* gene (position 10,833) and converts VP24 residue Lys163→Arg (**Figure 2d**). The wild-type residue, which is conserved among ebolaviruses, forms a salt-bridge with Asp104 in the Sudan virus VP24 crystal structure [23] (**Figure 3c**). However, Lys163 in the Reston virus VP24 structure [23] (not shown) points out in solution. With this mixed observation, we postulate that the VP24 variant has an insignificant effect on structural stability but may modulate interactions with other proteins.

Only one other SNP deletion was observed in the rest of the viral genome. This deletion occurred at position 13987, and it would result in a frameshift in the L ORF at amino acid position 802 and a truncated protein of 809 amino acids. This change was observed at a low level in all viruses (1.1 to 4.3%) and does not segregate between in vitro and in vivo passage (**Table 2**). Another significant SNP position was observed at position 8003 where all passages presented a minority population at significant levels (11.1 to 27.5%); however, this change would be silent and does not segregate between passages states (**Table 2**). Finally, variation in position 3,412 (<1.0 to 7.4%) of the genome leads to a change from Thr95 to Asn in VP35 decreased in vitro but did not have any reverting effect when infected into NHP (**Table 2**).

The significance of these findings is puzzling. It is highly unlikely that the observed variants arise spontaneously each time this virus changes environments, however, these viruses were not plaque purified during their historical passaging and we were unable to obtain genetic material from original clinical sample material. Thus, the selection forces behind the positive and purifying pressures are unknown. Previous work showed that the rEBOV-May/8U variant possessed a growth advantage over the rEBOV-May/7U variant and that it might enhance the rate of virion release *in vitro* [10]. Enhanced antagonism of tetherin, a type II transmembrane glycoprotein that inhibits the release of VP40-mediated virion-like particles (VLPs), was proposed to explain the early release of virions and the growth advantage [24]. Our data support this conclusion. Three independent lineages of EBOV-Kik passaged *in vitro* showed the same kind and type of mutations in the editing site. The rapid conversion of EBOV-May/8U to the wild-type EBOV-May/7U in guinea pigs after only one passage was interpreted as a sign that EBOV-May/8U may be incapable of efficiently replicating in the model as a result of its increased capacity of express GP_1,2_ and/or decreased expression of sGP. Increased cytotoxicity caused by GP_1,2_ overexpression; a decrease in sGP expression was also linked to a higher host clearance, because it has been postulated that sGP has a decoy function to confound the immune function. Our data confirm the general hypotheses, although the quick change to the 7U variant at day 8 after challenge might favor the cytotoxicity hypothesis rather than the decoy function.

The remaining change is located to the *VP24* gene. Further work to characterize this change and its effect on virus replication is needed. While these biomarkers seem to be ideal for identifying “in vitro”-grown EBOV-Kik, work would need to be completed with other circulating strains to determine if this set of SNPs is robust enough to use across several ebolaviruses and their various variants.

Our study has important implications for MCM as candidate vaccines or antivirals cannot be tested against highly lethal agents, such as filoviruses, in controlled clinical trials during a disease outbreak due to obvious ethical concerns. Therefore, the development of medical countermeasures against filoviruses relies on data accumulated from appropriate animal model studies and assessment by the Food and Drug Administration (FDA) under the 2002 “Animal Efficacy Rule” (FDA 21 CFR 601.90). Recently, the FDA has been encouraging standardization of filovirus animal experiments across agencies and institutes to ensure that results can be properly compared, making filovirus stock standardization a heavily discussed topic. Moreover, there is a large amount of interest in viral resistance development. However, the timeline for resistance development is very short during filovirus animal models (∼8–10 days). It then becomes very important to look at the viral minority population for resistance development. Understanding the selection pressures on the virus during in vitro and in vivo passaging would be critical to assess the mechanisms of selection during treatment. The results presented here confirm data presented by Volchkova et al. (22), namely that EBOV-Kik evolves into genomically different, but defined, subpopulations depending on whether they are administered to animals or cell culture. This observation raises several interesting questions: 1) Is cell-culture adaptation dependent on cell type, i.e., does 7A Ebola virus necessarily evolve to 8A Ebola virus in all cell types, or is this a Vero cell- or non-human primate cell-specific phenomenon? 2) While there are no substantial differences between animal experiments performed with EBOV-May and EBOV-Kik, our results demonstrate several differences between the putative wild-type virus (no complete genome is available) and the viral seeds. Would it be desirable to use an Ebola virus genomically as close to a wild-type, i.e.,unpassaged p0?. 3) What are the scientific explanations for the different evolution of Ebola virus subpopulations in cell culture versus animals? While the evolution to 8A Ebola viruses in animals can easily be hypothesized to be due to a yet-unidentified immunological selection process, it is less clear why a 7A Ebola virus would be preferred by individual cells in culture. 4) Can the results presented here be extrapolated to all ebolaviruses and are similar selection processes at work when marburgviruses, which do not contain an editing site, are passaged through animals and cells? Answers to these questions from scientists, in dialogue with FDA specialists, may influence how filovirus stocks will be prepared and characterized in the future (which cell line is to be used, how many passages are acceptable, which kind of sequencing will have to be performed at which stage?). They may also emphasize the need to accumulate more p0 filovirus genome sequences, of which there are currently few, for comparison studies.
